# Spontaneous Salivation Again

**Published:** 1863-12

**Authors:** Joseph A. Peters


### CORRESPONDENCE.

### SPONTANEOUS SALIVATION AGAIN.

Editor Medical and Surgical Journal:

The criticism of "Medicus" in the last Journal is written with so much apparent friendliness toward myself, that I can have no hesitation in accepting the writer's disclaimer of any intended personality, and hope to reply in the same spirit.

I am sorry he should have marred his article by the introduction of any slur upon the Medical Staff of the Army as a body; it is unworthy an appearance in so courteous an article. I shall enter into no defence of medical officers, as I do not consider it necessary; the bad ones I have no wish to shield, and the good ones do not need it.

"Medicus" criticises my article for not giving more detailed statements of the cases referred to, and hence assumes to doubt my memory, or my diagnosis, or both. Now in reply to this I can only say, that, during more than six months that I was in charge of the Regiment, I do not remember a case of ptyalism either with, or without mercury, except the few cases named, which all occurred in one camp, within a short time.—This circumstance served to fix my attention upon the subject, and led me to seek for some cause for the occurrence. As I was as certain as I could well be that some of my patients had taken no mercury, I was induced to believe some other agency was at work in these men. I expressly stated that none of them were otherwise healthy, all had more or less derangement of the digestive organs. Since my attention has been called to this subject, I have been told by one of our oldest and most scientific physicians, that he has often observed a similar affection in patients suffering from derangements of the *primæ viae,* and has been chagrined to find his patients salivated from some cause which he was unable to discover. He should have consulted "Medicus," who would have told him that it "depended upon an excess of acids," and would have quoted Braithwaite to him. "The acids naturally contained in the stomach are the muriatic and the acetic, and a grain or two of calomel or a few grains of blue mass would be sufficient to cause serious salivation, if thus changed into the bichloride."

There's a discovery for you! Chemistry. Materia Medica, Physiology and Therapeutics, in one easy lesson.

Can "Medicus" tell me by what process acetic acid can be made to convert a chloride into a bi-chloride? "No other paper has the news." But the best authority we have on the subject,* tells us that the only acid found in the free state in the gastric juice is lactic, though there are found of soluble chlorides about three and one-half parts in the thousand. It is to M. Mialhe that we are indebted for the theory that calomel, and other forms of mercury, are changed in the stomach (by the action of these chlorides) to the bi-chloride, yet he could only succeed in thus transforming one-fiftieth of a given weight of calomel by digesting for twenty-four hours, a much longer time than it usually remains in the stomach.

Again we have the practical fact, taught in the lecture room, and in text books, and observed by nearly all physicians that, of all the mercurials usually administered as medicines corrosive sublimate is most poisonous, but least efficacious in producing salivation.

Most, or all, of our text-books on the the practice of Medicine speak of an idiosyncracy which forbids the use of mercury, but our learned friend, by a single sweep of the feather end of his pen, dipped in an "excess of acids," banishes them forever from our view.

"Medicus's" system of Statistics is new and ingenious, it ought to have been promulgated to the "Statistical Congress" at Berlin at its late session. If, according to my theory, there were (as he says,) 5,000 cases of "spontaneous salivation" in the army, it is fair to conclude that, according to his, there were a like number afflicted with "an excess of acids." Had'nt somebody better abolish the acids?

I do not know that the precise dates are important, but as they have been alluded to I will say that all these cases occurred in one camp, and previous to August 9th, 1862. Some may have been seen in the latter part of July.

As to the literature of the question, one would conclude from Medicus's remarks that authors confine the disease to pregnant women and teething children. Yet Watson (Principles and Practice of Medicine, p. 529,) says, "Profuse pytyalism sometimes occurs without any obvious cause, and is then said to be *idiopathic* or spontaneous; and this is a circumstance it concerns you to be aware of, both as practitioners and as medical jurists. * * * But it is certainly of importance that you should be acquainted with the fact that ptyalism sometimes exists as a separate and independent malady." The same author also quotes (*loco citato*) several distin-

* Bernard, quoted by Dalton.

guished authorities to the same effect, one of whom (Dr. Pereira) had seen a dozen cases. Wood and others mention it also.

"Medicus" says somewhat cavalierly, "To detect the difference between mercurial and spontaneous salivation would not, I fancy, be very difficult for an experienced physician."

In the case of "pregnant women and teething children," this is true, I believe; but in the spontaneous salivation under discussion it is not so true. In the former case we have absence of fœtor, a marked symptom, but in regard to the latter, Watson says, we have "The same *tenderness and swelling* of the salivary glands, the same copious *secretion and excretion of saliva,* nay, even the same *fœtor,* or a smell which can scarcely be distinguished from it." I do not believe even "Medicus" could distinguish the mercurial from the non-mercurial affection without knowing the history of the case; I know I could not, and I only claimed to *think* myself right, admitting the possibility of error, while he assumes I am wrong on mere hypothesis.

The "obloquy" to which I referred did not come from Surgeon-General Hammond, but from the

"Blanche, Tray and Sweetheart"

of the newspapers who have recently turned their wrath against General Hammond himself; I am rejoiced to see, however, he has come off triumphant from his trial.

As I do not know "Medicus," (wherein he has the advantage of me) I hope he will not need to be told that I entertain no pique towards him.—Nevertheless, if he will meet me over a dose of the "most effectual," with our respective meerschaums, I shall be pleased, while he is explaining the *modus operandi* of changing a chloride to a bi-chloride by means of acetic acid, to tell him by what process I was enabled to "put mercury out of the question"—if we are not both lost in a cloud of our own raising.

Yours, truly, JOSEPH A. PETERS.

